# The prevalence of attention-deficit hyperactivity disorder and its associated factors among children in Ethiopia, 2024: a systematic review and meta-analysis

**DOI:** 10.3389/frcha.2024.1425841

**Published:** 2024-08-23

**Authors:** Molla Azmeraw, Dessie Temesgen, Amare Kassaw, Alemu Birara Zemariam, Gashaw Kerebeh, Gebremeskel Kibret Abebe, Addis Wondmagegn Alamaw, Biruk Beletew Abate

**Affiliations:** ^1^ Department of Nursing, College of Health Science, Woldia University, Woldia, Ethiopia; ^2^ Department of Pediatrics and Child Health Nursing, College of Health Science, Debre Tabor University, Debre Tabor, Ethiopia

**Keywords:** ADHD, children, meta-analysis and systematic review, Ethiopia, adolescent

## Abstract

**Introduction:**

Attention-deficit hyperactivity disorder (ADHD) is a neuropsychiatric condition that affects children. Its magnitude varies by area, ranging from 0.2% to 26.8%. Even though there is debate, culture and geographical location may have little or no influence on the epidemiology of ADHD worldwide. Despite this variation, debate over the national prevalence and location of ADHD is unknown in Ethiopia. Therefore, this study aimed to assess the pooled prevalence of ADHD and its contributing factors among children.

**Methods:**

Electronic databases, including Google Scholar, PubMed, Scopus, EMBASE, Web of Science, ScienceDirect, and institutional repositories, were searched. The studies that covered the prevalence and/or risk factors of ADHD in children were included in the collection. The Joanna Briggs Institute quality rating tool was used to rate the quality of each study. The data were extracted using Microsoft Excel 2019, and the statistical analysis was performed using STATA 17.0. Using a random-effects model, we evaluated the combined prevalence of ADHD and associated factors. The Cochrane *Q*-test and *I*^2^ test statistics were used to quantify the heterogeneity. Furthermore, publication bias was examined using funnel plot graphs and Egger's tests. A Galbraith plot was employed to illustrate outliers. Sensitivity analysis was also applied.

**Result:**

This study included a total of six articles with 4,338 participants. The pooled prevalence estimate of ADHD was 8.81% [95% confidence interval (CI), 4.52–13.11; *I*^2^ = 96.95%; *P* = 0.001]. Age 6–12 [adjusted odds ratio (AOR) = 3.51 (95% CI, 1.38–5.64), *I*^2^ = 0%; *P* = 0.001], being male [AOR = 1.94 (95% CI, 1.09–2.79), *I*^2^ = 0%; *P* = 0.001], and living with a single parent [AOR = 4.92 (95% CI, 1.24–861), *I*^2^ = 0%; *P* = 0.001] were significant variables.

**Conclusion and recommendation:**

One out of every 12 children in Ethiopia suffers from ADHD. Living with a single parent, being male, and being between the ages of 6 and 12 were risk factors for ADHD. A nationwide study with a large sample size may be required to ascertain the true impact of ADHD. It may be crucial to improve school health services to identify ADHD early and lessen its long-term effects.

## Introduction

Attention-deficit hyperactivity disorder (ADHD) is a neuropsychiatric disorder that occurs during childhood. It is a clinically heterogeneous neurodevelopmental syndrome that comprises developmentally inappropriate inattentiveness, hyperactivity, and increased impulsivity (1).

The diagnosis of ADHD is not based on a single pathogenic alteration. Although the precise cause of ADHD has not been established, it is mostly brought on by a decrease in frontal brain activity (2). It is diagnosed by the presence of certain behaviors. It was the 98th leading cause of years lived with disabilities (YLDs) for people across all ages and the 84th for males across all ages (3). Within each of the three childhood age groups, ADHD was ranked as the 52nd, 44th, and 61st leading cause of global YLDs (3). In males aged 10–14 years, it was the 34th highest contributor to global YLDs, coming in ahead of diabetes, meningitis, and intellectual disability (4). Children and adolescents with ADHD can be measured using a variety of established standard instruments. A number of instruments are used to measure ADHD, including the Disruptive Behavior Disorder Rating Scale (DBDRS) (5), Conners–Wells Adolescent Self-Report Scale (CASS) (6), Diagnostic Interview for Child and Adolescents-Revised (DICA-R) (7), Swanson, Nolan, and Pelham rating scale fourth revision (SNAP-IV-C) (8), Vanderbilt ADHD Teacher Rating Scale (VARTRS) (9), ADHD rating scale (10), and Diagnostic and Statistical Manual of Mental Disorders, Fifth Edition, Text Revision (DSM-V) (11).

The prevalence of ADHD varies from 0.2% in Germany using participant interviews among children aged 12–17 years to 26.8% in Brazil using a teacher's questionnaire among children aged 6–15 years (12, 13). Another global study estimated that approximately 5% of children and adolescents are affected by ADHD (14). A systematic review and meta-analysis study in Africa also showed that the estimated pooled prevalence of ADHD was 7.47% (15). In addition, a review report showed that ADHD among African children ranged from 5.4%–8.7% (16). Similarly, in Uganda, children with ADHD accounted for 2.67%–6% of the study participants (17–20). In other East African countries, such as Sudan, the estimated prevalence of ADHD has been reported to be 10.6% and 9.4% (21, 22). The estimates of ADHD prevalence in Ethiopia range from 1.5% to 13.7% (23, 24). Moreover, a previous study revealed that conduct disorder (CD) and ADHD contributed a total of 6.24 million YLDs/disability-adjusted life years (DALY) to the total global burden of disease (25). There is a gender difference in ADHD prevalence, with a high male-to-female ratio reported in several studies (15, 26, 27) except for a study report that showed a higher proportion of ADHD in females than males (28). In contrast, other previous studies in Ethiopia and Germany reported an equal female to male ratios (29, 30). The global prevalence studies also support a higher proportion of ADHD in males than in females (31–33).

ADHD is caused by several factors, including genetic and environmental factors. Brain pathology, such as head trauma, is another commonly reported risk factor for ADHD (16). Family history of mental illness, substance abuse, age, pregnancy-related complications, premature birth, preeclampsia, hypertension, excess weight, obesity in pregnant women, maternal smoking exposure, low socioeconomic status, and the existence of psychosocial stressors have also been described as risk factors for ADHD (19, 26, 27, 34, 35). In the Ethiopian context, five subnational studies reported at least one factor associated with ADHD in Ethiopia (26, 27, 34–36). Financial crises (26, 27, 34), children's history of mental disorders (34), cesarean section (C/S) delivery (34), lifetime substance abuse (34), maternal complications during pregnancy (26, 35), illiteracy of the mother (35), attending primary school (35), history of head trauma (35), maternal alcohol use during pregnancy (35), bottle feeding during the first 6 months (35), age of the child (26, 35, 36), having a single parent (27), presence of a chronically sick family member (36), being male (26, 27), child birth order/rank (27), having a family size greater than five (26), presence of one or more psychosocial stressors (26), and family history of mental illness (26) were the reported factors significantly associated with ADHD (26).

ADHD can have a significant impact on patients and their families at every stage of life (37). As children with ADHD make the transition from childhood to adolescence to adulthood, changes in the symptoms of the disorder tend to occur. It has been observed that hyperactive and impulsive symptoms of ADHD decrease but inattentive symptoms remain (38). A previous study observed that children with ADHD were at a higher risk of psychiatric problems, substance abuse, low self-esteem, social problems, difficulty (a greater chance of being rejected by his or her peer group), criminal justice issues, academic failure (increased delinquency and grade repetition, reduced future reading and mathematics scores, and an increased likelihood of receiving special education), poor work performance, and disturbed family functioning in adolescence than non-hyperactive children (39, 40). In addition, a quality-of-life study among children and adolescents diagnosed with ADHD showed a significant impact of ADHD on multiple domains of health-related quality of life. ADHD-affected children and adolescents struggle with things like staying still, organizing their space, and focusing while working. These and other symptoms frequently make it difficult for them to deal with the school environment, which has an adverse effect on relationships and prospects for work in the future (32). In general, ADHD may significantly impair multiple aspects of life, leading to educational underachievement, unemployment, unsuccessful marriage, and criminality (41, 42). In addition, ADHD shows significant correlations with a wide range of comorbid psychiatric disorders, including affective disorders, defiant disorder, antisocial personality disorder, self-harm, and substance misuse, placing a considerable burden on society and the family. The Diagnostic and Statistical Manual of Mental Disorders (DSM) and the International Classification of Diseases (ICD) describe ADHD (“hyperkinetic disorder” in the ICD) as a disruptive disorder characterized by persistent hyperactivity, impulsivity, and/or inattention (25). According to these criteria, a case must first occur before the age of 7 (43). Studies suggest that ADHD imposes a significant economic burden on society across multiple domains of direct, indirect, education, and justice system costs for both children and adults with ADHD (44).

Currently, the international community has given priority to the prevention of mental health problems, including ADHD. The fourth target of the United Nations’ Sustainable Development Goal 3 has also promised to “reduce premature mortality from non-communicable diseases by one-third and promote mental health and well-being by 2030” (45). The World Health Organization has launched a new campaign to improve mental health in keeping with Sustainable Development Goal 3. In the Ethiopian context, in an effort to provide all citizens with accessible, acceptable, and reasonably priced mental health services, the Ethiopian government has taken these international initiatives on board and incorporated them into its national health sector strategic documents, such as the health system transformation plan, mental health strategy, and health extension programs (46). A mental, neurological, and substance abuse disorder intervention guideline was developed by the World Health Organization. To design a parallel national intervention guideline, baseline data on the status of each prioritized mental illness is important. Moreover, as described before, the estimated prevalence of ADHD varies from country to country, depending on the diagnostic criteria used, the tools used for measurement, and the function of geographic and cultural perspective variations. As far as our knowledge allows, even though ADHD is one of the behavioral disorders in children and adolescents, its national status is unknown. The Cochrane Collaboration and the evidence-based medicine movement are the driving forces behind systematic reviews and meta-analyses. Compared with cross-sectional studies, they have advantages in terms of bias reduction, replicability, reliability, dispute resolution, and a trustworthy basis for decision-making. Therefore, to settle the ongoing disputes between studies conducted at the subnational level across the country, a systematic review and meta-analysis were employed to determine the estimated pooled prevalence of ADHD. The prevalence reports of the previous studies were inconsistent, and there are disagreements on the risk factors that exposed children to ADHD. Therefore, this study aimed to assess the prevalence of ADHD and its associated factors among children in Ethiopia using existing literature.

## Research question

What is the national prevalence of attention-deficit hyperactivity disorder among children in Ethiopia?

## Objective

### General objective

To assess the prevalence and associated risk factors of attention-deficit hyperactivity disorder among children in Ethiopia, 2023.

### Specific objectives

To estimate the pooled prevalence of attention-deficit hyperactivity disorder among children in Ethiopia, 2023.

To determine the risk factors correlated with attention-deficit hyperactivity disorder among children in Ethiopia, 2023.

## Methods

### Study design and setting

According to study design, every study on ADHD carried out in Ethiopia was cross-sectional. The prevalence reports of the previous studies were inconsistent, and there are disagreements on the risk factors that exposed children to ADHD. Furthermore, compared with systematic reviews, cross-sectional research generates less evidence. Therefore, to settle the ongoing disputes between research conducted in the nation, a systematic review and meta-analysis were employed to determine the pooled prevalence of ADHD. The description of Ethiopia has been summarized elsewhere (47).

### Searching strategy

Preferred Reporting Items for Systematic Reviews and Meta-Analyses (PRISMA) guidelines were used to conduct this study (48). To access articles, we employed both systematic searches using electronic databases (such as Google Scholar, PubMed, EMBASE, Web of Science, ScienceDirect, Psych INFO, and Scopus) and manual searches using institutional repositories (including a hand search of the reference lists of the included studies). The following search phrases and keywords were used to find pertinent papers across all databases: Medical Subject Headings term combinations were used with the key terms attention-deficit hyperactivity disorder, prevalence, determinants, children, adolescents, and Ethiopia to create effective search methods.

These key terms were combined using the Boolean operator “AND/OR” to narrow the search in the databases. The search term in combination with the Boolean operator “[prevalence OR Rate** OR Proportion OR epidemiology OR magnitude) AND [“attention deficit hyperactivity disorder” OR ADHD] AND [child**] AND [Ethiopia]” was used in PubMed and Google Scholar. Some databases, such as Psych INFO, EMBASE, Science Direct, and SCOPUS, were searched using database-specific subject headings using the above keywords like “attention deficit hyperactivity disorder OR hyperkinetic disorder OR ADHD AND children OR child OR childhood AND Ethiopia”. No date limit was applied. The reference lists of the included studies were also screened for the presence of additional studies. This systematic review and meta-analysis study was not registered with PROSPERO. The last search was made on 11 December 2023 in all databases and PROSPERO.

### Eligibility criteria

#### Inclusion criteria

In general, the criteria for selecting documents in the systematic review and meta-analysis were within the framework of PICO (Population, Intervention, Comparison, Outcomes) (49). In this study, the eligibility criteria were based on these conditions.

**Population:** all people under the age of 18 were examined in this study.
**Exposure:** ADHD-affected children and adolescents.
**Outcomes:** because of the differences in diagnostic criteria for ADHD, the prevalence of ADHD was evaluated using DSM-V, DSM-IV, DSM-IV-TR, and ICD10 criteria.
**Design**/**type of study:** cross-sectional observational studies were used to establish the disease's prevalence.
**Setting:** studies conducted in Ethiopia were included.
**Language:** only English language publications were included. In this regard, online language translation was considered as a last option if an article was published in a language other than English.
**Publication year:** all articles published before 28 November 2023 were included.

#### Exclusion criteria

Articles about reviews, editorials, non-human subjects, and those that omitted information about prevalence and/or associated factors were excluded. The titles and abstracts were screened using the prespecified inclusion and exclusion criteria before the retrieval of full-text articles for further screening. Two reviewers (BA. and MA) independently performed the screening. In the second step, the two reviewers independently read the full-texts of the articles that were not excluded in the initial stage and met the inclusion criteria. When there was any disagreement between two authors, the dispute was resolved by consensus or after discussing it with a third reviewer.

### Methods for data extraction and quality assessment

Two authors, AK and MA, separately extracted all the data from the included research articles. The data extraction form used by the authors was specifically created to produce data for this systematic review and meta-analysis. Summary tables with information on authors, publication year, study setting, study design, study population, response rate, sample size, number of outcomes, percentage, and ADHD assessment instruments were created by extracting data from the included studies. Information from the included studies was extracted in line with the assessment template prepared as recommended by PRISMA guidelines.

For evaluating quality, we employed the Joanna Briggs Institute (JBI) critical appraisal instrument. Three writers (MA, BA, and GK) independently evaluated the quality of the included studies. For scores of less than 50%, 50%–75%, and more than 75%, we classified the overall quality scores as low quality (high risk of bias), moderate quality (moderate risk of bias), and high quality (low risk of bias), respectively (50). The appraisal instrument had the subsequent eight criteria: (1) inclusion criteria; (2) study subject and context description; (3) valid and reliable exposure measurement; (4) standard and objective criteria applied; (5) confounder detection; (6) confounder handling techniques; (7) outcome measurement; and (8) appropriate statistical analysis. When a study met 50% or more of the criteria on the quality evaluation checklist, it was deemed low risk (50, 51). The result of the quality assessment is available in the Supplementary Material.

### Definition of outcome (ADHD)

The outcome definition was given based on DSM diagnostic criteria.

### Data synthesis and analysis

We conducted a meta-analysis using a random-effects model for the studies that provided appropriate statistical data to determine pooled estimates of ADHD prevalence and 95% confidence intervals (CIs). Data extraction was performed using Microsoft Excel 19 and analysis was carried out using STATA 17.0 statistical software. We used the Q and *I*^2^ statistics to evaluate heterogeneity. The fraction of total variance among the included studies that went into creating the observed heterogeneity was evaluated by the *I*^2^ statistics. True homogeneity in this study is indicated by an *I*^2^ statistic value of zero, while values of 25%, 50%, and 75% represent moderate, medium, and high heterogeneity, respectively. Subgroup analysis was taken into consideration for the data that were found to be heterogeneous. A leave-one-out sensitivity analysis was performed to assess the important studies that have a significant influence on the heterogeneity between studies. Egger's regression test and funnel plots were used to evaluate publication bias. *P* < 0.05 analyses were considered significant.

## Results

### Study selection

**Figure 1:**
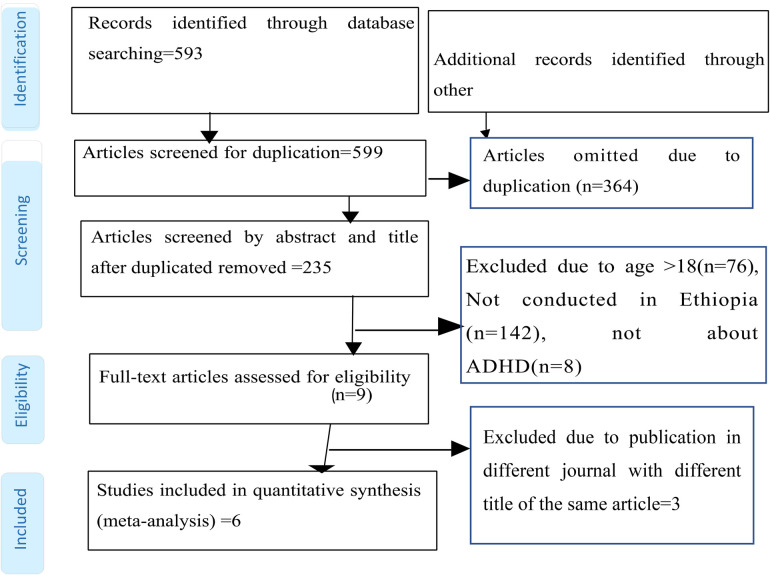
PRISMA flow diagram showing the selection of studies to estimate the pooled prevalence of ADHD among children in Ethiopia, 2023.

Through the use of electronic searches (via database searching, *n* = 599), 599 studies were found up until 28 November 2023. The EndNote reference manager program, version X8, was used to manage the duplicate research among the accessed publications, along with a manual comparison of titles and abstracts. After combining all references from all databases and using the “find duplicates” feature in the “reference” menu bar of the EndNote program version X8, the duplicate references were removed. After removing duplicates, there were 235 articles overall (364 duplicates). Subsequently, 235 studies were selected for full-text review based on their title and abstract. Eight papers did not address ADHD, 76 were removed because the participants were over the age of 18, 142 were removed because they were not conducted in Ethiopia, and 3 (23, 24, 30, 36, 52) were excluded because they were due to publish in a different journal with a different title for the same article. Quality checks were performed on the remaining nine items. Three further papers were eliminated because they were published in a different journal under a different title with the same authors. Finally, six articles, including a total of 4,338 study participants, met the quality assessment criteria (Figure 1). The authors took these into account when analyzing the estimated prevalence and/or determining related factors.

### Characteristics of the studies

Six studies were included in this meta-analysis and systematic review (Figure 1). The features of the included studies are explained clearly in Table 1 (23, 24, 26, 27, 34, 35). The two studies conducted by Tiruneh (24) and Murugan and Tiruneh (52) were similar. As the study was conducted by Tiruneh and is available in the Addis Ababa University thesis and dissertation portal, we used the article by correspondent author Tiruneh. Similarly, three published articles by Ashenafi et al. (23, 30, 36) were conducted in south nation nationalities and people's region, Butajira, in 1998. Therefore, the authors used one of them for analysis. In addition, all studies were cross-sectional in study design, with one institutional study and five community-based studies by setting. The studies were published from 2001 to 2023. The study participants in the included studies ranged from 355 (34) to 1,447 (23) (Table 1). The population age distribution of the study participants in the included studies was nearly similar, i.e., 6–15 (one study), 6–16 (one study), 5–15 (one study), and 6–17 (three studies). Although Ashenafi et al., Lola et al., and Aliye et al. did not report it, the mean age ranged from 9.56 ± 2.63 (26) to 10.99 ± 3.04 (34). The reported prevalence of ADHD ranged from 1.5% (23) up to 13.7% (24) across the included studies (Table 1). The JBI quality assessment tool was used. The quality assessment results of all the included studies yielded 6–8 out of eight questions asked, which corresponded to a 75%–87.5% quality standing (Supplementary Table S1).

**Table 1:** Characteristics of the included studies conducted on the prevalence of ADHD among children in Ethiopia, 2023.

Reference	Study type	Region	Design	Age ranges (years)	Mean age	Sample size	Outcome (*N*)	Prevalence (%)	Quality
Ashenafi et al. (23)	CB	SNNPE	CS	5–15	—	1,447	22	1.5	8
Mulu et al. (34)	CB	Amhara	CS	6–17	10.99 ± 3.04	355	46	13	6
Lola et al. (27)	CB	Oromia	CS	6–17	—	1,238	90	7.3	8
Benti et al. (26)	IB	Oromia	CS	6–15	9.56 ± 2. 63	407	34	8.4	7
Tiruneh et al. (24)	CB	Oromia	CS	6–16	9.83 ± 2.77	387	53	13.7	6
Aliye et al. (35)	CB	Oromia	CS	6–17	—	504	50	9.9	7

CB, community based; CS, cross-sectional; IB, institution based.

### Proportion of ADHD subtypes

This study finding showed that some authors reported the subtypes of ADHD (26, 27, 34). The highest and lowest inattentive type ADHDs were reported by Lola et al. (63.3%) (27) and Benti et al. (0.7%) (26). Benti et al. (5.2%) (26) and Mulu et al. (25%) (34) also reported that there was a higher proportion of a combined type of ADHD than the other type (Table 2).

**Table 2:** Proportion of ADHD subtypes in studies conducted on the prevalence of ADHD among children in Ethiopia, 2023.

Inattentive type (%)	Hyperactive/impulsive (%)	Combined (%)	Tool
_	_	_	Diagnostic Interview for Children and Adolescent (DICA)
10.80%	14.15	25	Disruptive Behavior Disorder Rating Scale
63.30%	24.50%	12.2	Disruptive Behavior Disorder Rating Scale
0.7	2.5	5.20%	Disruptive Behavior Disorder Rating Scale (DBD rating scale)
18.1	14.5	—	Disruptive Behavior Disorder Rating Scale (structured parent rating scale)
—	—	—	Vanderbilt Attention Deficit Hyperactivity Disorder-Parent Rating scale as a modified, semi-structured, and face-to-face interview

### Prevalence of ADHD

**Figure 2:**
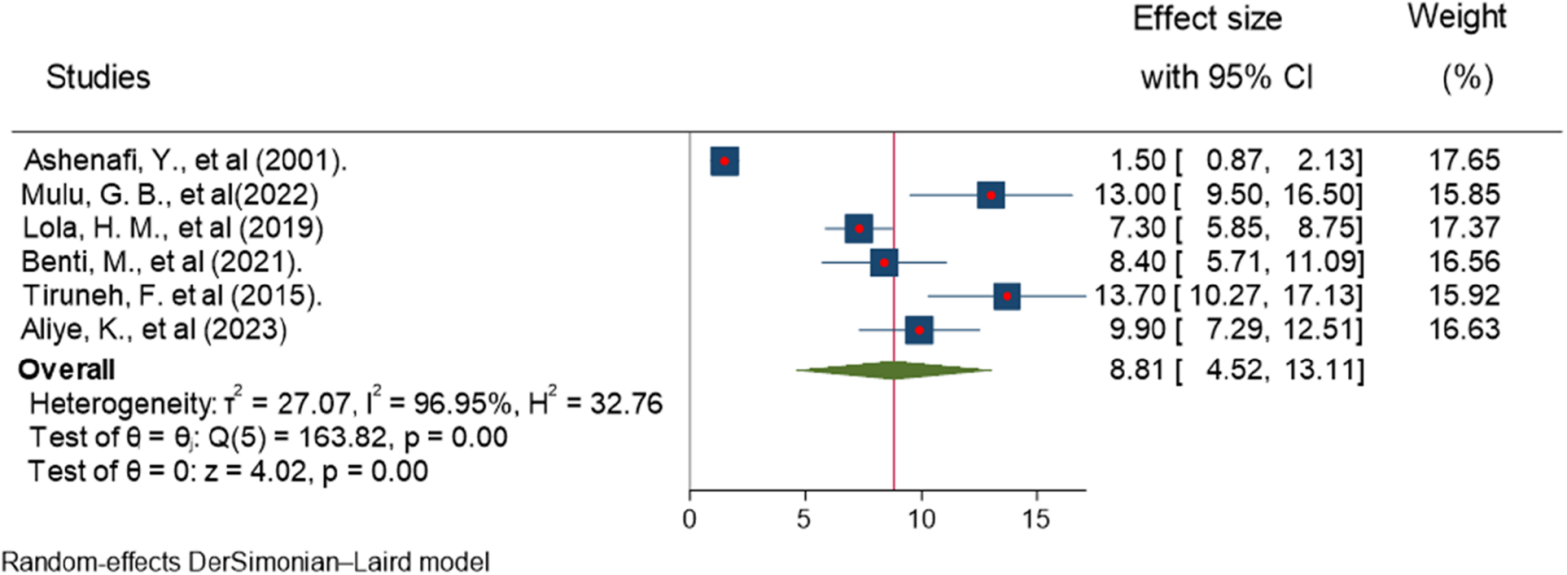
Forest plot showing the estimated pooled prevalence of ADHD among children in Ethiopia, 2023.

All of the included studies reported estimates of ADHD prevalence. The random-effects model analysis of these studies revealed that the pooled estimate of ADHD prevalence was 8.81% (95% CI, 4.52–13.11; *I*^2^ = 96.95%; *P* = 0.001) in Ethiopia. A high heterogeneity (*I*^2^ = 96.95%; *P* = 0.001) was observed (Figure 2).

### Subgroup analysis

The subgroup analysis was carried out based on sample size and region. The studies with sample sizes larger than 1,000 individuals reported the highest pooled estimates of ADHD prevalence. Similarly, the Amhara region has the highest frequency of ADHD, followed by the Oromia region (Table 3).

**Table 3:** Subgroup analysis of the studies by region and sample size.

Subgrouping variable	Categories	Heterogeneity (*I*^2^, *p*-value)	Prevalence of ADHD with 95% CI
Sample size	355–1,000	61.37%, *p* = 0.05	11.04 (8.61–13.47)
	>1,000	98.07%, *p* = 0.001	4.36% (1.32–10.05)
Region	Amhara	0.00%, *p* = 0.001	13% (9.5–16.5)
	Oromia	76.15%, *p* = 0.01	9.53% (7.04–12.02)
	SNNPE	0.00%, *p* = 0.001	1.5% (0.87–2.13)

### Sensitivity analysis

**Figure 3:**
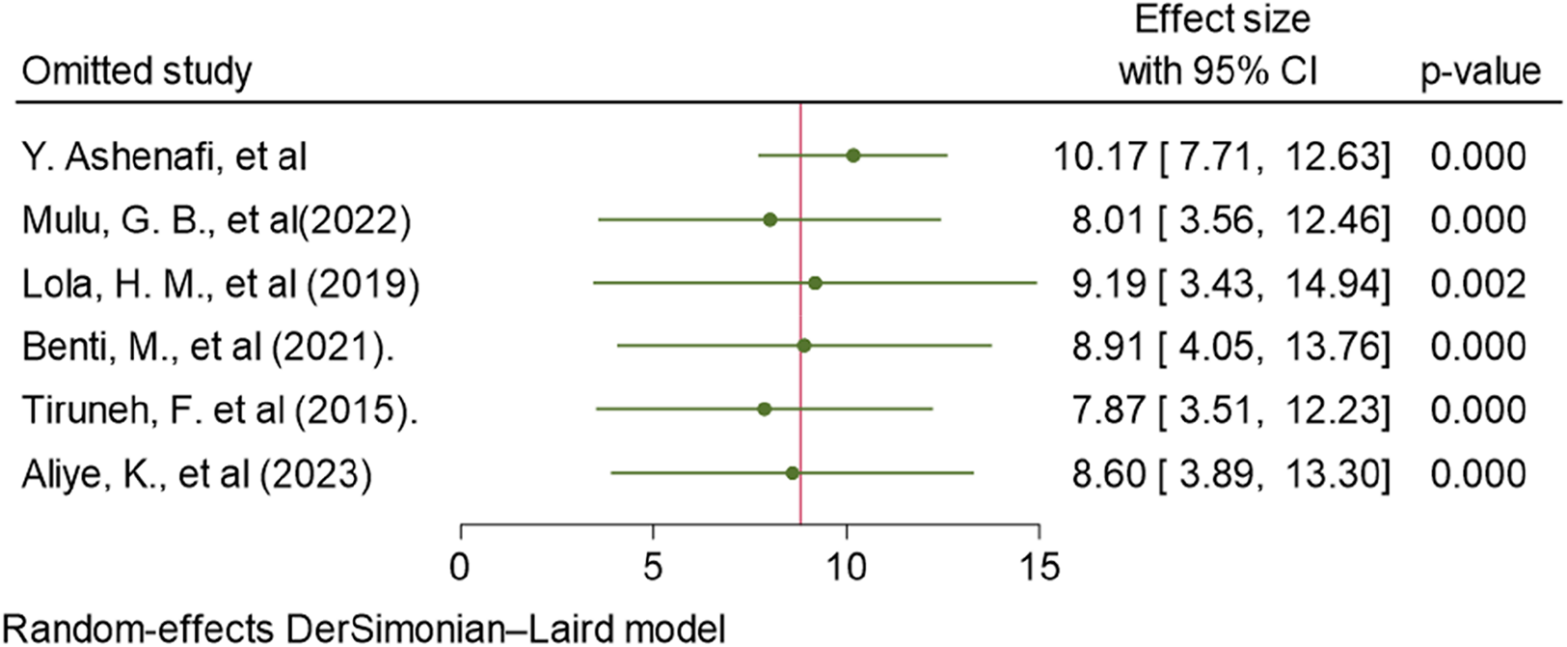
Sensitivity analysis of the included studies in estimating the pooled prevalence of ADHD in Ethiopia, 2023.

To find the possible presence of outliers or influential studies in the analysis of the estimates of ADHD prevalence in the Ethiopian sample population, a leave-one-out sensitivity analysis was conducted. The outcomes demonstrated that the conclusions were not reliant on a single study, and no influential study was detected. The estimated range for the pooled estimates of ADHD prevalence was 7.87 (95% CI, 3.51–12.23) (24) and 10.17 (95% CI, 7.71–12.63) (23) after the deletion of a single study (Figure 3). In addition, the homogeneity assumptions were tested by checking the randomness of one study at a time. The *P*-value of the Q-statistics was significant for all studies randomness once at a time, which indicates the presence of heterogeneity.

### Publication bias

**Figure 4:**
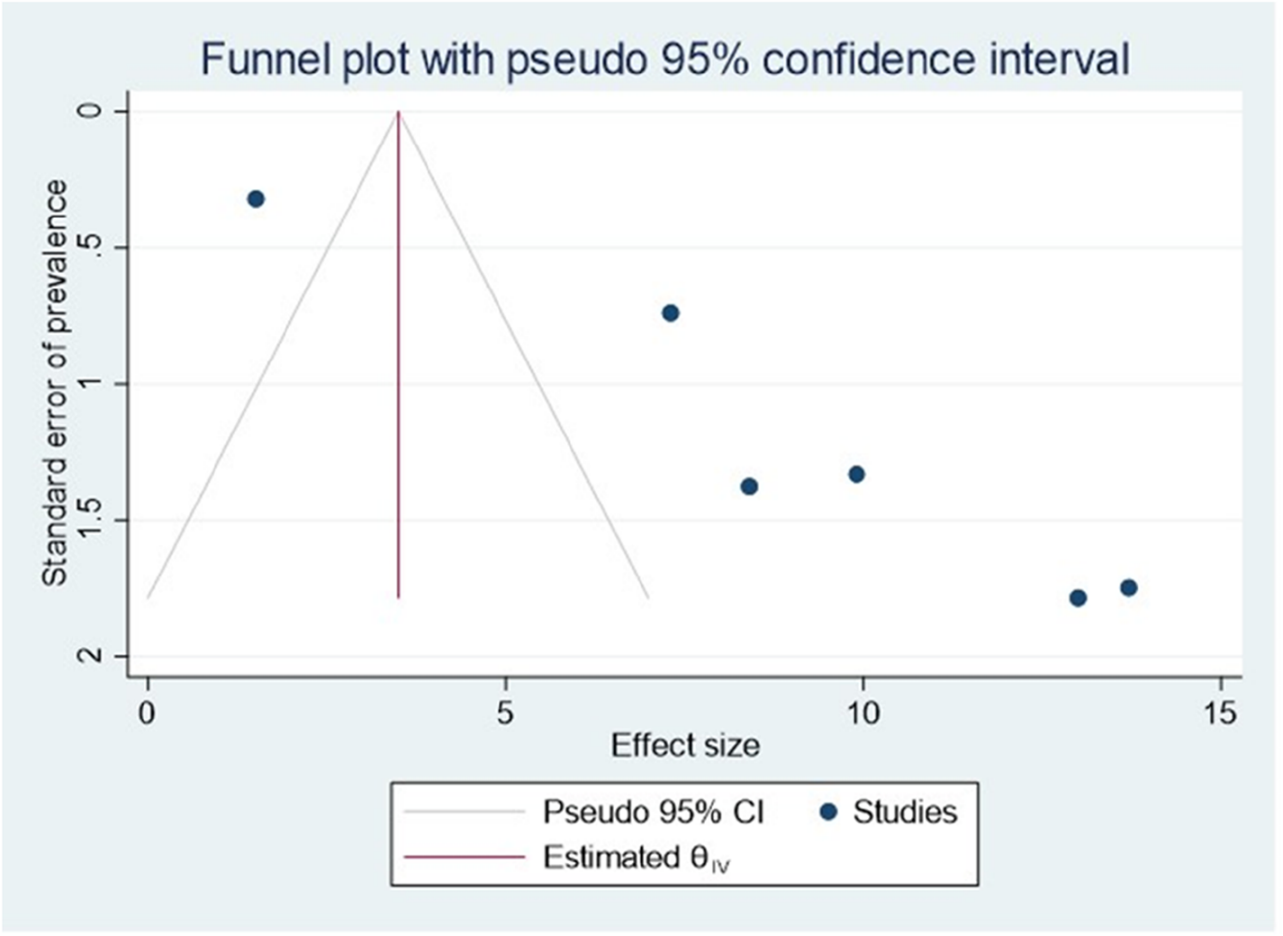
Funnel plot showing evidence of publication bias when estimating the pooled prevalence of ADHD among children in Ethiopia, 2023.

Publishing bias was investigated using a funnel plot, which suggested a bias in the publications (Figure 4). The *P*-value for Egger's statistic was 0.001, which further supported the existence of publication bias.

**Figure 5:**
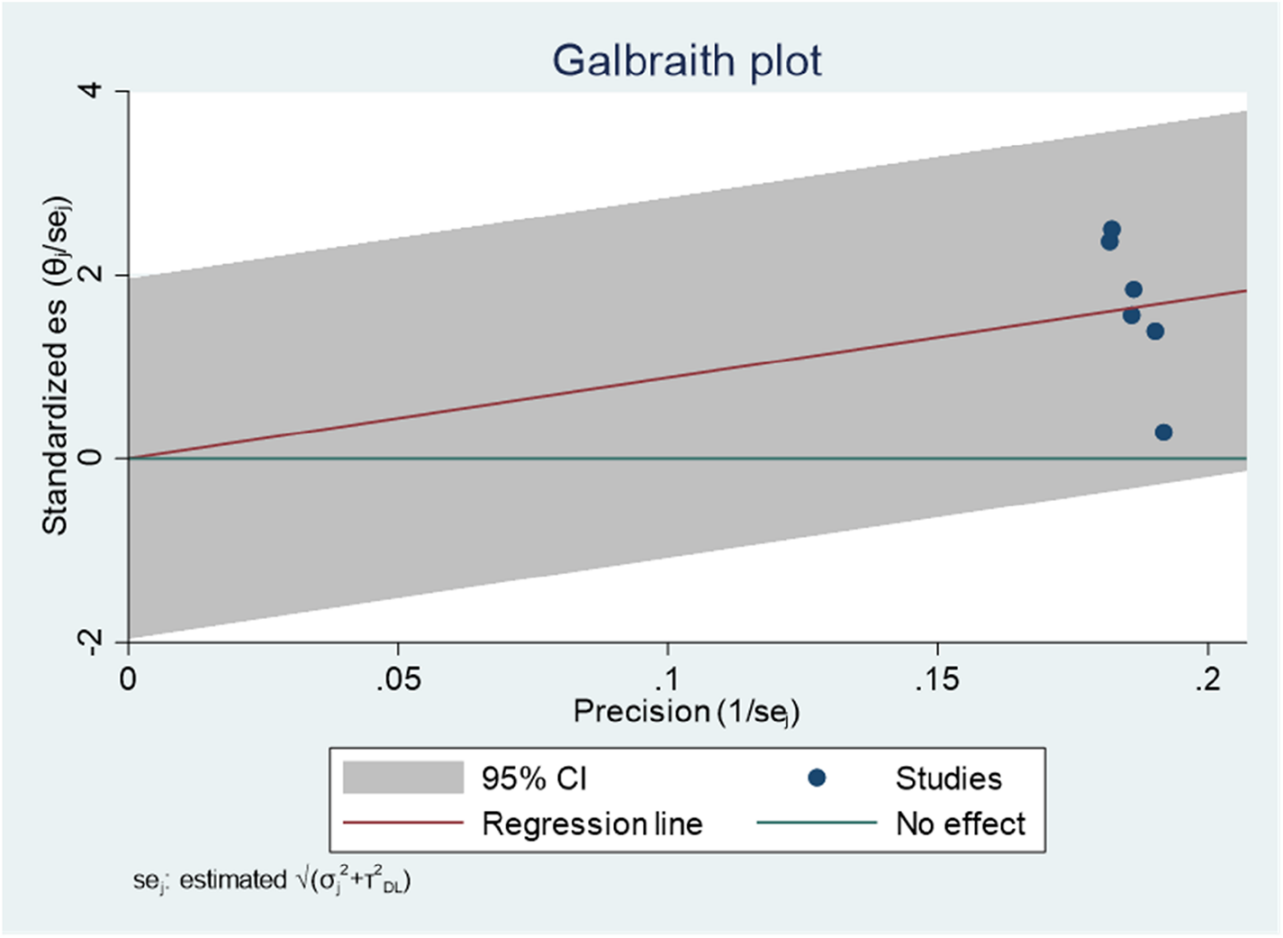
Galbraith plot showing the outlier studies on ADHD in Ethiopia, 2023.

The Galbraith plot graph clearly shows outlier studies (Figure 5). Studies conducted by Mulu et al. (34) and Tiruneh et al. (24) were responsible for the discrepancy.

### Trim-and-fill analysis

**Figure 6:**
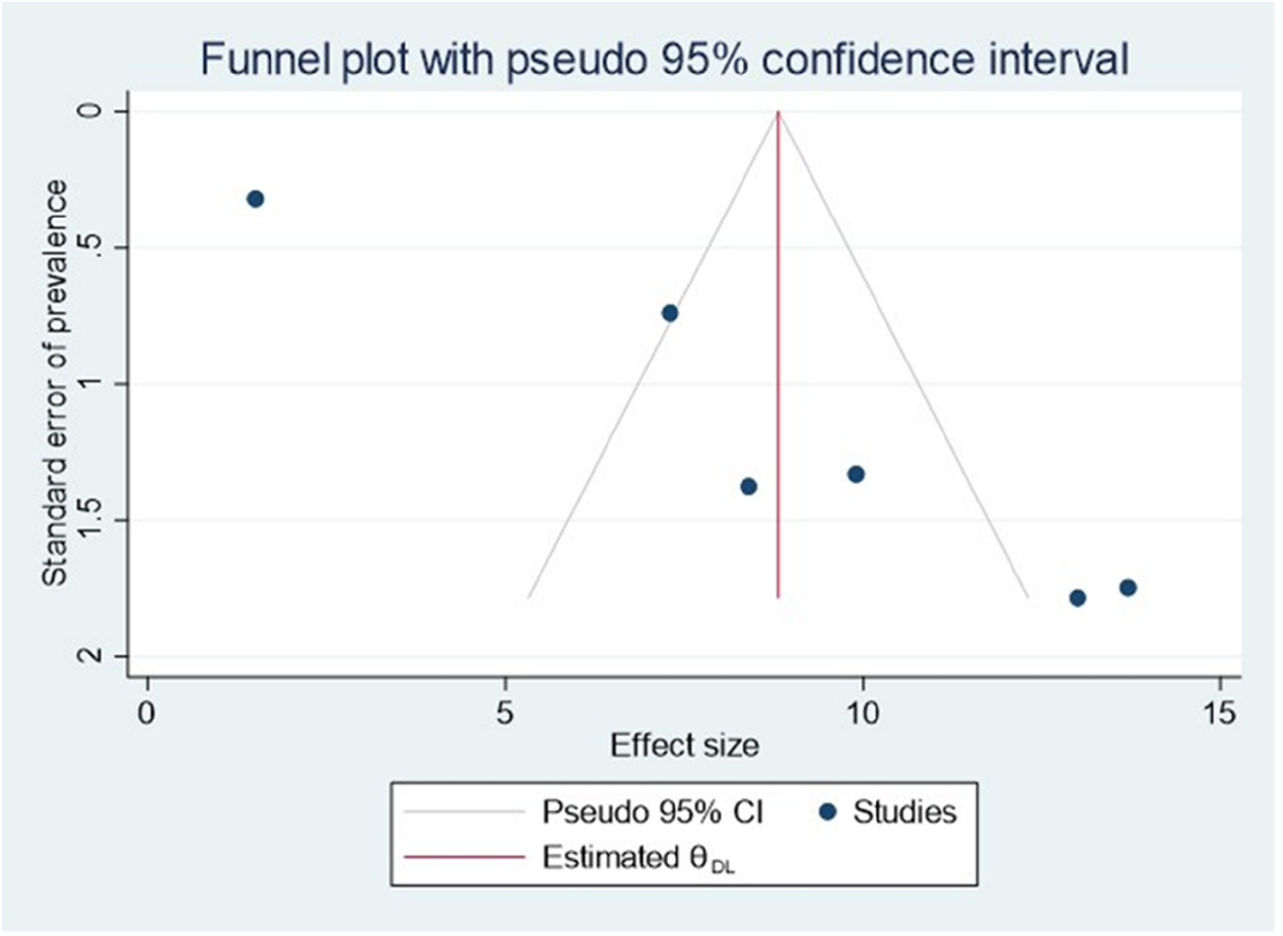
Funnel plot of trim-and-fill analysis for estimating the pooled prevalence of ADHD among children in Ethiopia, 2023.

The non-parametric trim-and-fill analysis of publication bias was employed. There is no imputed article and difference in effect size (Figure 6).

### Factors associated with ADHD

Five of the included studies reported at least one factor associated with ADHD in Ethiopia (26, 27, 34–36). Financial crises (26, 27, 34), children's history of mental disorders (34), C/S delivery (34), lifetime substance abuse (34), maternal complications during pregnancy (26, 35), illiteracy of the mother (35), attending primary school (35), history of head trauma (35), maternal alcohol use during pregnancy (35), bottle feeding during the first 6 months (35), child's age (26, 35, 36), having a single parent (27), the presence of a chronically sick family member (36), Being male (26, 27), having a child birth order or rank ( 2), having a family size greater than five (26), the presence of one or more psychosocial stressors (26), and having a family history of mental illness (26) were the reported factors significantly associated with ADHD (26). The pooled estimate of being aged under 12 years, living with a single parent, and being male were significant risk factors of ADHD in children. A detail description of these significant factors was given hereafter.

### Age

**Figure 7:**
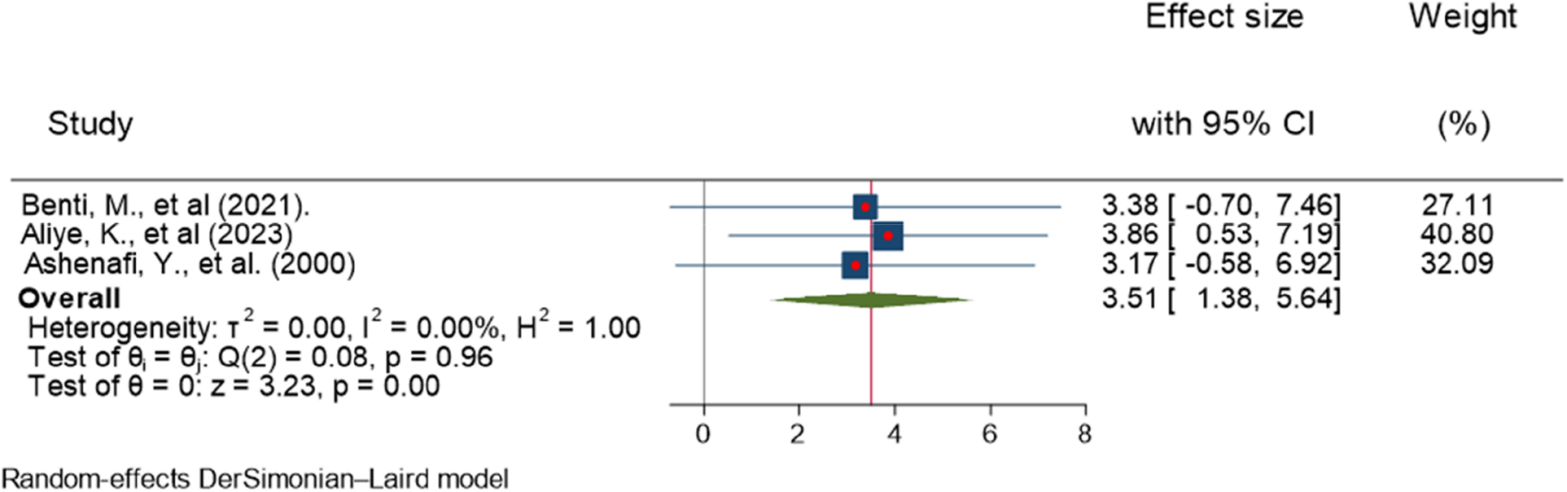
Forest plot showing the pooled AOR of the association between age and ADHD among children in Ethiopia, 2023.

Aliye et al. (35), Ashenafi et al. (36), and Benti et al. (26) reported a significant association between age and ADHD. The forest plot showed that the overall estimate of the adjusted odds ratio (AOR) for children aged 6–12 years was 3.51 (95% CI, 1.38–5.64); *I*^2^ = 0; *P* = 0.001), which meant 6–12-year-old children were 3.51 times more likely to develop ADHD than 13–17-year-old children. The *I*^2^ value and *P*-value showed the homogeneity of the studies (Figure 7).

**Figure 8:**
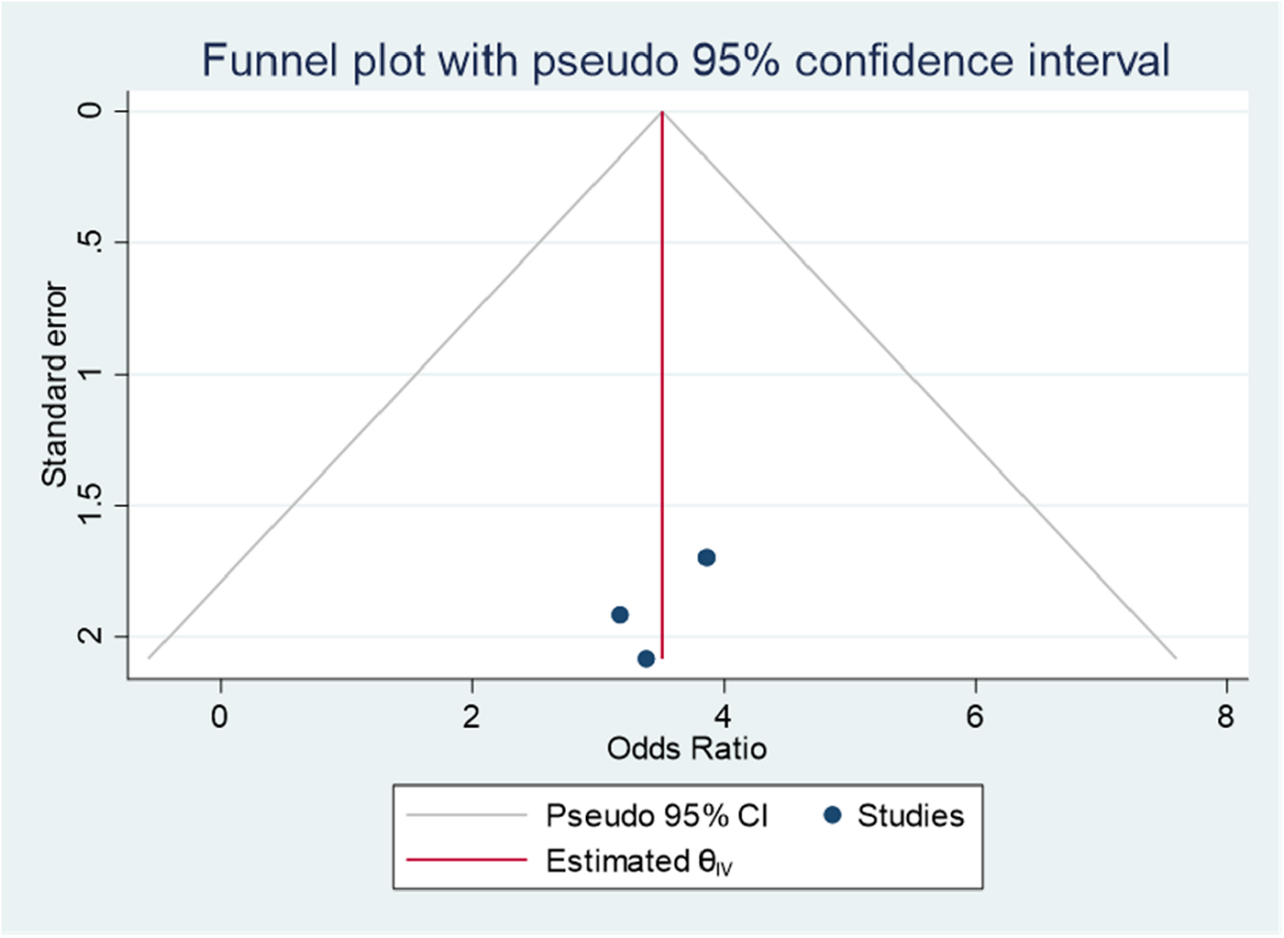
Funnel plot showing evidence of publication bias when estimating the AOR of the association between age and ADHD among children in Ethiopia, 2023.

**Figure 9:**
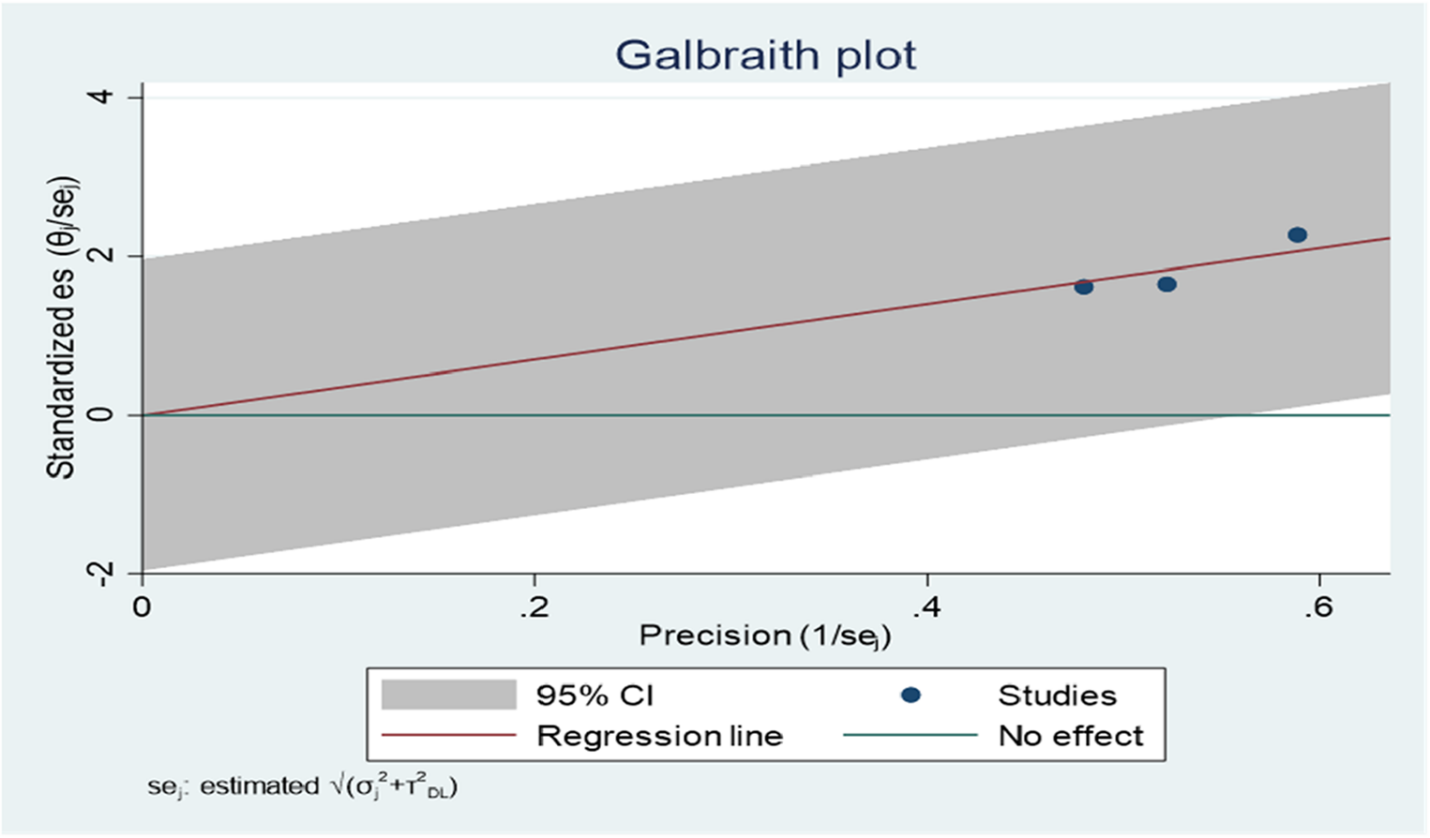
Galbraith plot showing the outlier studies for estimating the AOR of the association between age and ADHD among children in Ethiopia, 2023.

Regarding publication bias, the funnel plot analysis showed a symmetrical distribution (Figure 8). During the Egger's regression test, the *P*-value was 0.8296, which indicated the absence of publication bias. The Galbraith plot demonstrated the absence of outlier studies (Figure 9).

### Sex

**Figure 10:**
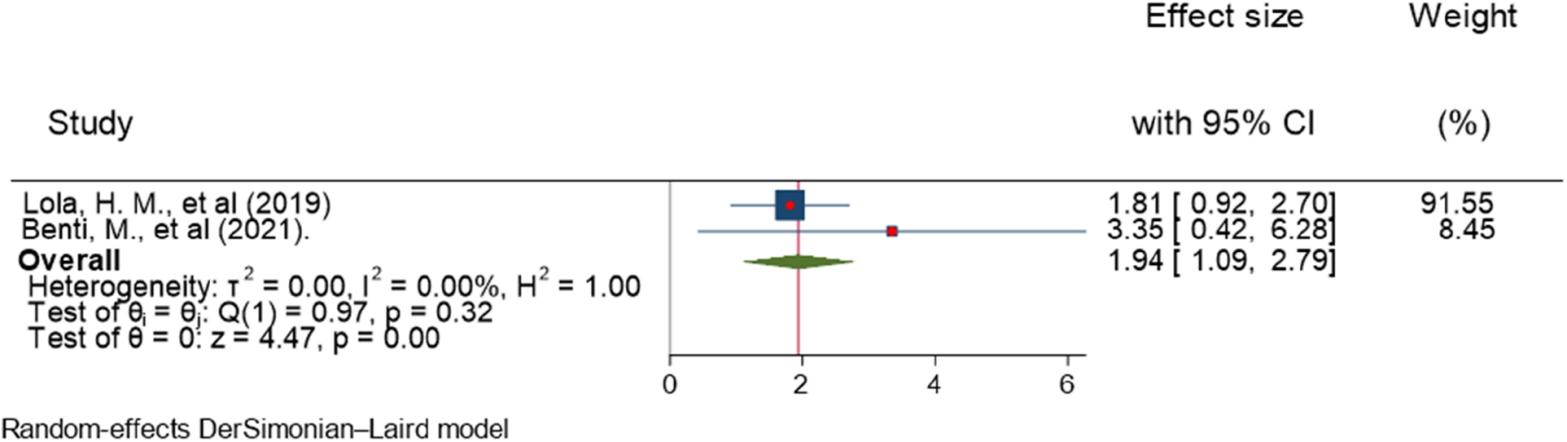
Forest plot showing the pooled AOR of the association between sex and ADHD among children in Ethiopia, 2023.

Lola et al. (27) and Benti et al. (26) reported a significant association between sex and ADHD. The forest plot combined result of these studies showed that the overall AOR estimate of being male was 1.94 (95% CI, 1.09–2.79; *I*^2^ = 0%; *P* = 0.001). Males were 1.94 times more likely to have ADHD than females. The *I*^2^ value and *p*-value showed the homogeneity of the findings (Figure 10).

**Figure 11:**
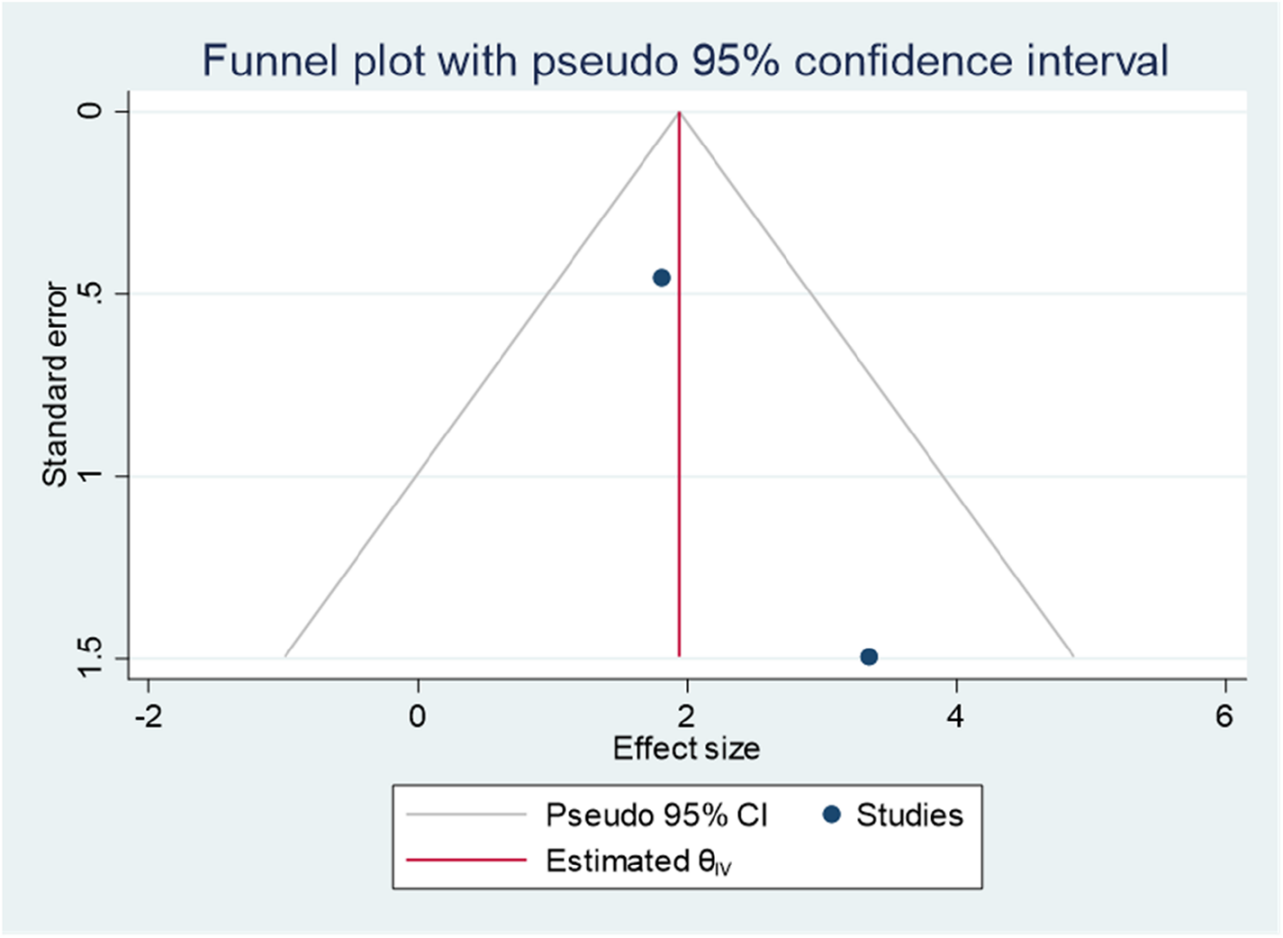
Funnel plot showing evidence of publication bias when estimating the AOR of the association between sex and ADHD among children in Ethiopia, 2023.

**Figure 12:**
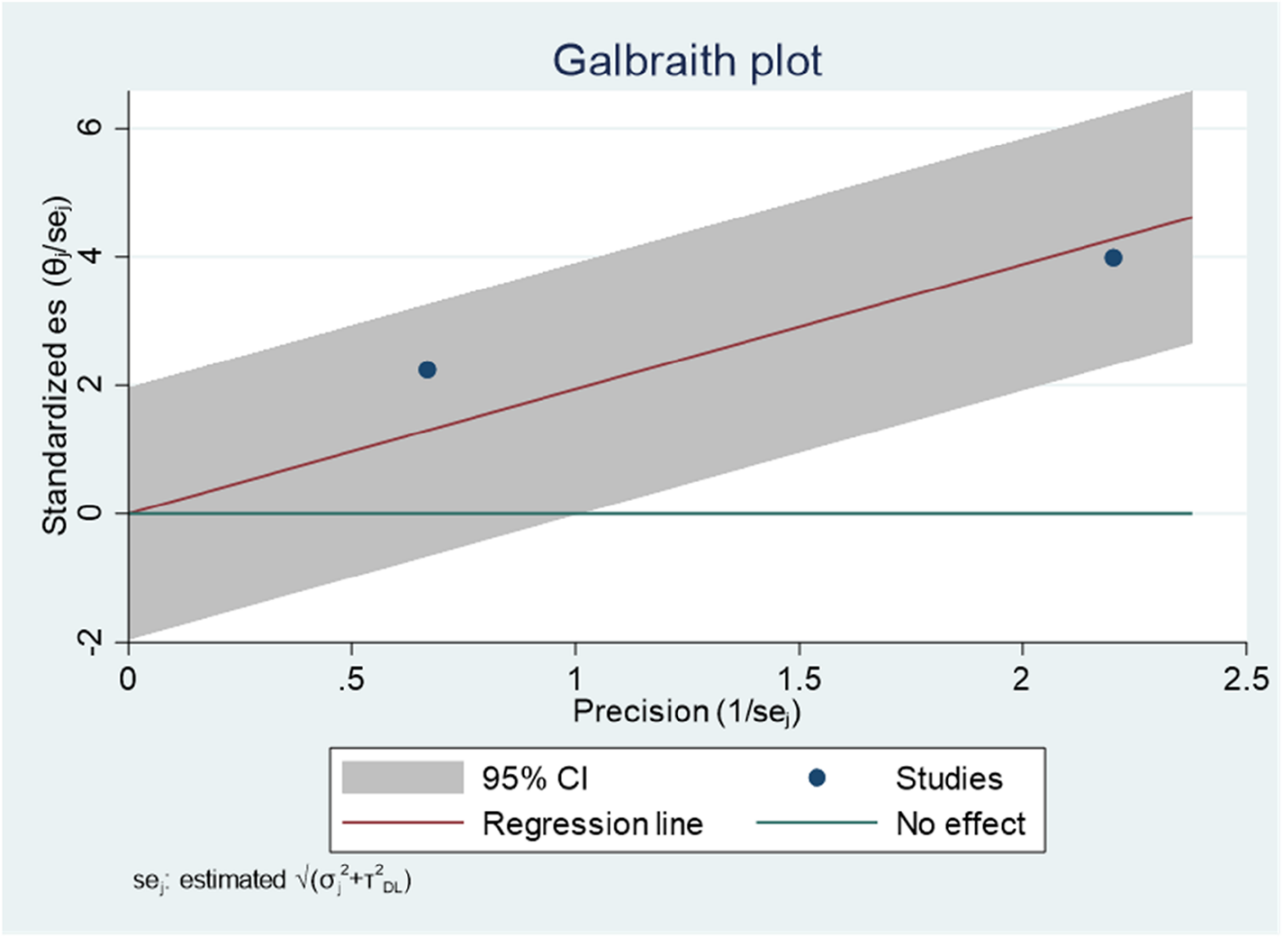
Galbraith plot showing the absence of outlier studies when estimating the AOR of the association between sex and ADHD among children in Ethiopia, 2023.

Regarding publication bias, the funnel plot showed a symmetrical distribution (Figure 11). Egger's statistical test supported the graph visualization of the absence of publication bias, with a *P*-value of 0.3243. In addition, the Galbraith plot showed the absence of outlier studies (Figure 12).

### Single parent

**Figure 13:**
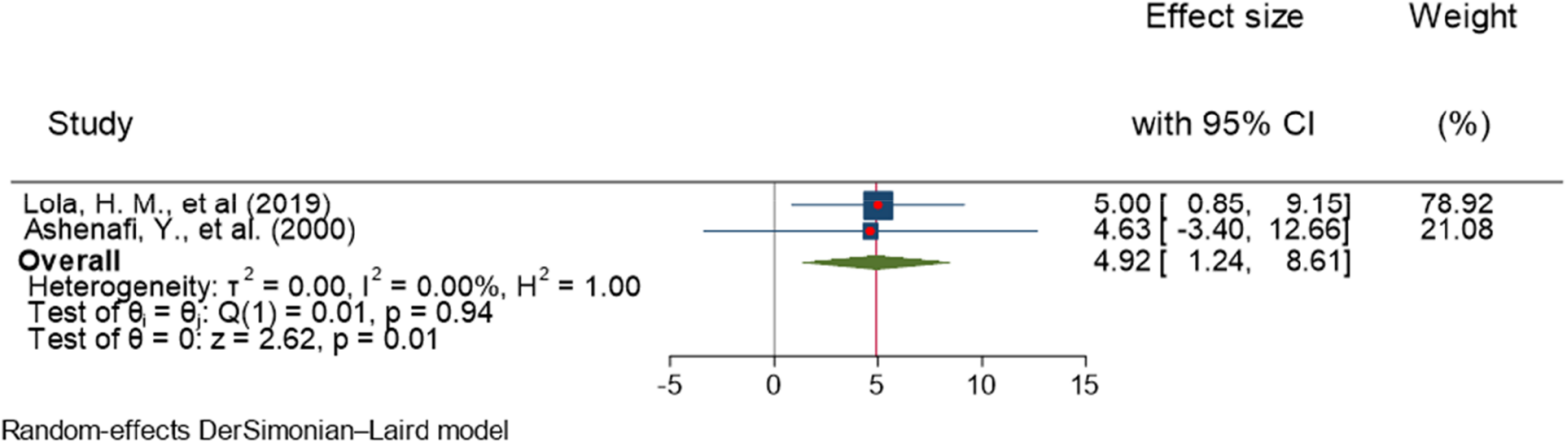
Forest plot showing the pooled AOR of the association between living with a single parent and ADHD among children in Ethiopia, 2023.

Lola et al. and Ashenafi et al. reported a significant association between single parents and ADHD among children (27, 36). The combined result of the two studies on a forest plot presented an overall estimate of AOR of 4.92 (95% CI, 1.24–8.61; *I*^2^ = 0.0%; *P* = 0.001) (Figure 13). The *I*^2^ value and *P*-value showed homogeneity. The odds of ADHD was 4.92 times more likely in children living with a single parent than those living with both parents.

**Figure 14:**
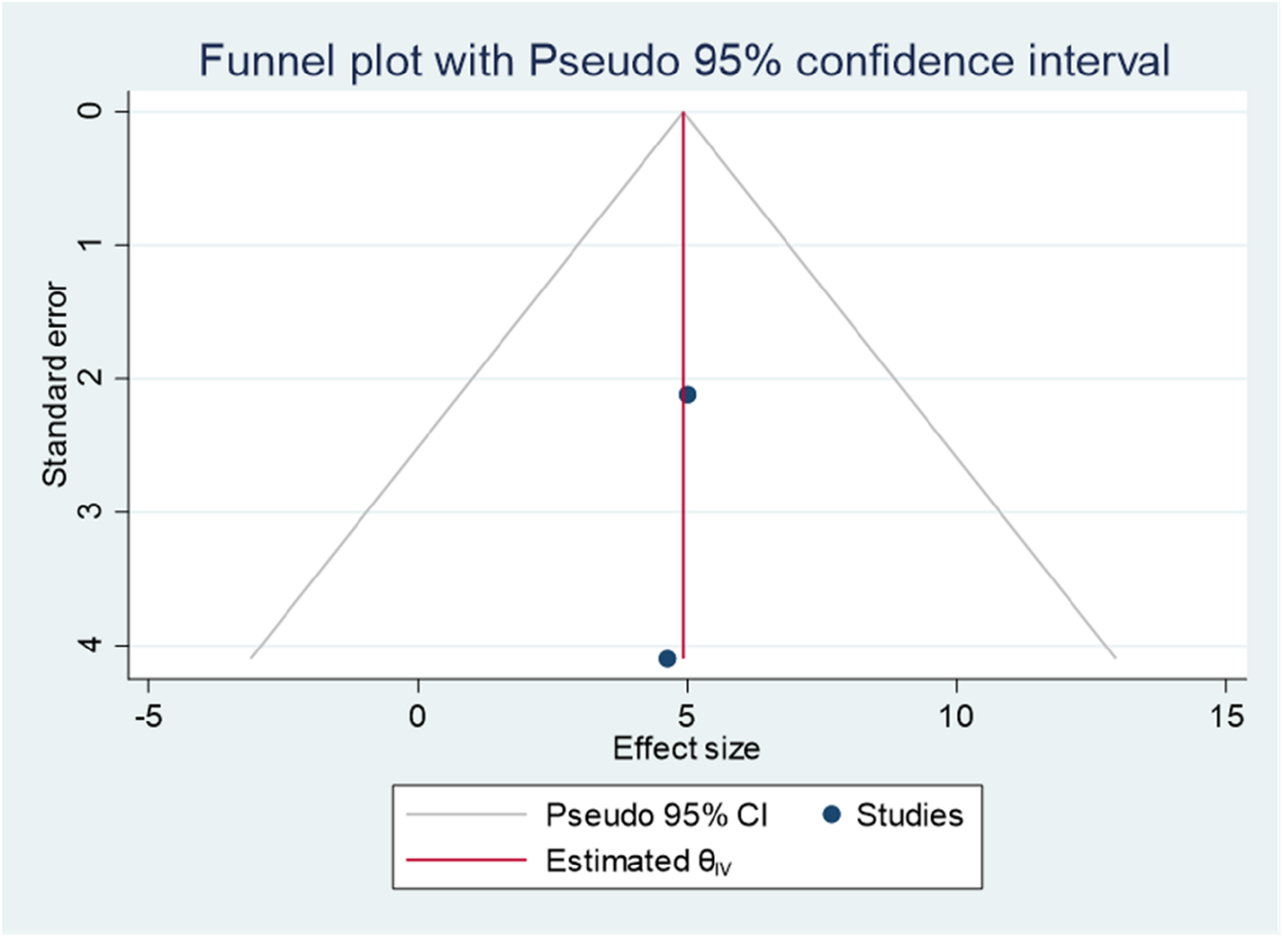
Funnel plot showing evidence of publication bias when estimating the AOR of the association between living with a single parent and ADHD among children in Ethiopia, 2023.

**Figure 15:**
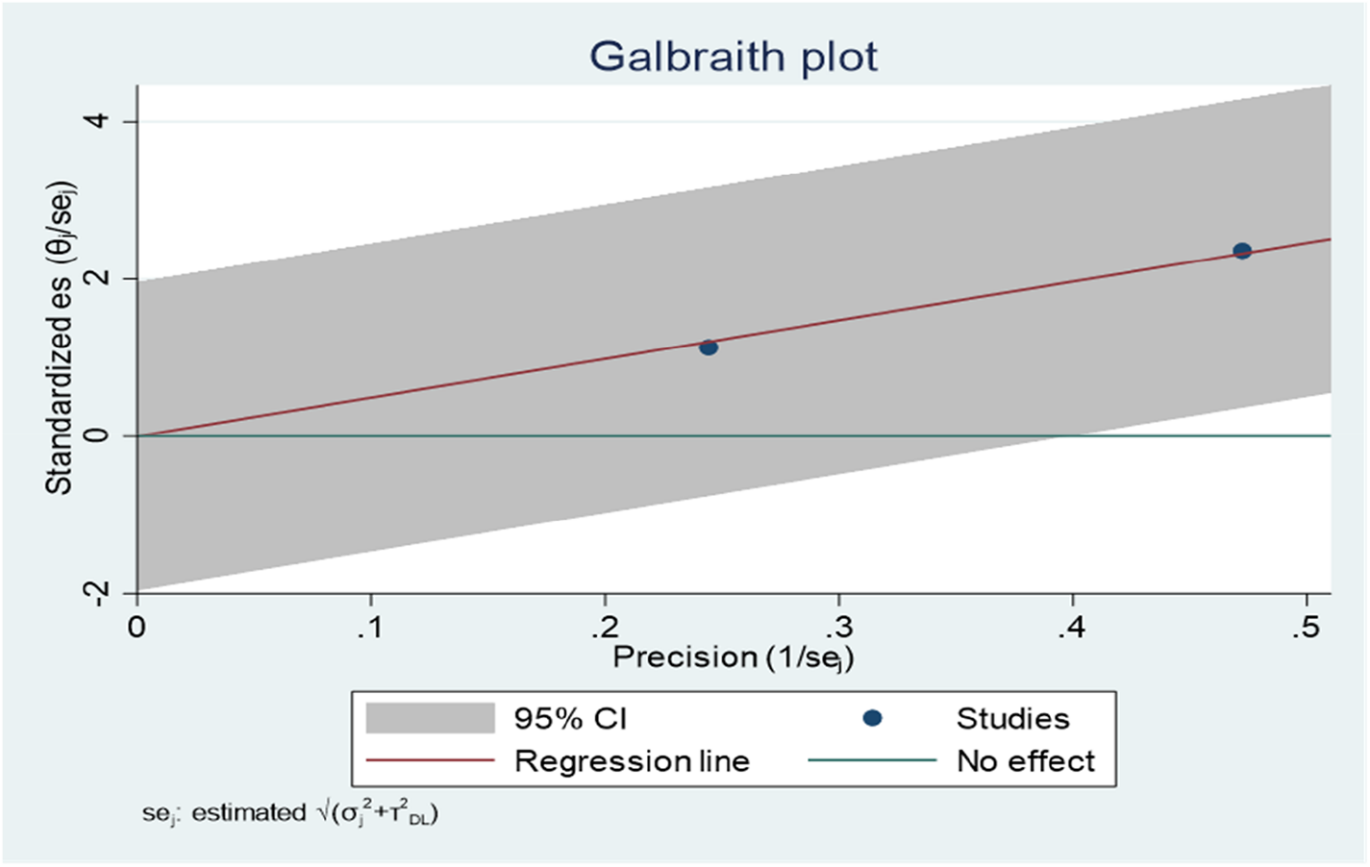
Galbraith plot showing the outlier studies for estimating the AOR of the association between living with a single parent and ADHD among children in Ethiopia, 2023.

Regarding publication of bias, the funnel plot analysis showed a symmetrical distribution (Figure 14). During the Egger's regression test, the *P*-value was 0.9372, which indicated the absence of publication bias. In addition, the Galbraith plot did not show any outliers (Figure 15).

## Discussion

This systematic review and meta-analysis study included six studies, published between 2001 and 2003, to assess the pooled prevalence estimates of ADHD in Ethiopia. The authors believe that this systematic and meta-analysis study is the first of its kind regarding ADHD in Ethiopia among children. The subnational ADHD prevalence estimates varied from 1.5% to 13.7% (23, 24) in Ethiopia. The lowest record was in 2001 and the highest ADHD prevalence estimate was reported in 2015. The discrepancy might be because of the fact that it was not due to a real increase in prevalence but rather to improved diagnosis or an increase in functional impairment (53). In addition, the variation might be due to the population, setting (community-based vs. institution-based), method of assessment, diagnostic criteria employed, source of information, and inclusion of impairment in functioning in operational definition criteria (16). In this study, the pooled prevalence estimate of ADHD was 8.81%. Several reviews of the literature have reported highly variable prevalence rates of ADHD worldwide, ranging from 0.2% in Germany using participant interviews among children aged 12–17 years to 26.8% in Brazil using a teacher's questionnaire among children aged 6–15 years (14). This finding was in line with other previous, national, subnational, and global studies, such as in China (7.5%) (54), the USA (8.7%) (55), Spain (6.8%) (56), Iran (7.2% and 8.7% in different areas) (57, 58), Africa (5.4%–8.7%) (15, 16), and globally (5.9%–7.6%) (59–61). However, the finding was lower than those reported by other previous studies, which reported ADHD prevalence of 15.5% (USA) (62), 16.1% (Colombia) (63), 17.9% (Brazil) (64), 19.8% (Ukraine) (65), 19.7% (South Africa) (66), and 18.9% (USA) (67). In addition, this review finding was also higher than in previous studies conducted in Iran (3% and 4%) (68, 69), India (1.6%) (70), Russia (1.3%), and Spain (4.06%) (71). The prevalence rate difference, whether higher, lower, or in line, might be due to the diagnostic criteria used (DSM-III, DSM-IIIR, DSM-IV, DSM-V, or ICD-10), method of diagnosis (questionnaire or interview), characteristics of the population (male, female, age), number of pieces of information used (parents only, teacher only, or combined), comorbidity (inclusion or exclusion of cases with a comorbid diagnosis), setting [community-based or school-based (higher prevalence reported in community-based studies than in school-based studies)], and socioeconomic differences (high prevalence in low socioeconomic groups) (72). Studies using DSM criteria that include criteria for impairment, pervasiveness, and comorbidity reported a high prevalence of ADHD (72). The specific cause of ADHD is unknown. There is still an unclear pathophysiology for ADHD. Genetic and environmental exposures play a pivotal role in the occurrence of ADHD (54, 73). Dopamine, serotonin, and norepinephrine are the main neurotransmitters involved in the pathophysiology of ADHD (40). It is predominately associated with decreased activity in the frontal lobe. In children with ADHD, specific neuroimaging abnormalities were observed on the frontal subcortical cerebral pathway, which is involved in the control of attention, inhibition, and motor behavior (72).

There is a gender difference in the ADHD subtype distribution report in estimates of ADHD prevalence. This study finding showed that some authors reported the subtypes of ADHD (26, 27, 34). The highest and lowest levels of inattentive type ADHD were reported by Lola et al. (63.3%) (27) and Benti et al. (0.7%) (26). Benti et al. (5.2%) (26) and Mulu et al. (25%) (34) also reported a higher proportion of a combined type of ADHD than the other types. In Iran, the combined ADHD subtype was the most prevalent (44%), the hyperactive-impulsive subtype was second (38%), and the inattentive subtype was third (17%). This subtype pattern was the same for boys; however, the hyperactive-impulsive subtype was more prevalent in girls (63%) (58).

In this study, age, sex, and living with a single parent were significantly associated with ADHD. The odds of ADHD among males are higher than their counterparts. This can be due to a variety of causes, such as referral bias, variations in comorbidity patterns, the effects of hormonal shifts, and variations in the way symptoms present in males and females. These could result in female ADHD patients receiving an incorrect diagnosis or being underdiagnosed, delaying or preventing therapy. Girls with ADHD can be identified and treated earlier if parents, educators, and medical professionals are more aware of these differences and can identify them. Most of the time, the hyperactive-impulsive type of ADHD is more common in males than females, and the inattention type is more common in females (74). As a result, male ADHD symptoms are more likely to be presented externally, whereas female ADHD symptoms are often internalized and are therefore not as noticeable. What readers also need to understand is that ADHD was first defined based on the behavior of hyperactive boys, and much of the available assessment still focuses on external behaviors. This finding implies that male schoolchildren require a greater focus than female schoolchildren. On the other hand, underdiagnosis and overdiagnosis of ADHD have been reported (2). Tools used to measure ADHD may need validation for these problems in both sexes.

ADHD is more prevalent in children aged 6–12 years than in children aged 13–17 years. This finding was consistent with previous studies (75–78). The discrepancy across the age groups might be due to the continuous reduction of the core symptoms over the lifespan. In general, only approximately 5%–15% of patients continue to completely fulfill the diagnostic criteria for ADHD in adulthood, although persistent symptoms or functional impairment remain in approximately 70% of them; however, findings vary widely across studies due to methodological differences and other reasons (53). The symptoms of ADHD are detected at around 3 years of age and have a tendency to continue into adolescence and adulthood. It is more common in school-age children than adolescents (72, 79, 80). This study finding implies the need for ADHD screening for children during their school age period (6–12 years). In addition, a further study is required to determine whether there is an age cluster of ADHD or not. Those children living with a single parent were more likely to have ADHD than those living with both parents. This finding is consistent with studies in Sweden (81) and Egypt (82).

This might be due to the fact that a lack of time and money are more common features of the everyday life of a single parent. This may lead to a lack of social support and family conflict, including divorce, separation, and parental absence (81). Research has demonstrated that children raised by lone parents are more susceptible to mental health issues, addiction, and attempted and actual suicide (83). Living in a stressful environment can manifest as altered physiological function, such as dysregulation of the hypothalamus, pituitary, and adrenal cortex (HPA) axis (84). It might also raise the possibility of developing behavioral and mental health symptoms. The quality of parent–child relationships in a family is also impacted by single parenthood, which has long-term implications for the risk of children and youths engaging in antisocial behavior (85). This implies that a special emphasis needs to be placed on single parent children with regard to mental health problems, including ADHD.

Even though this study revealed that age, sex, and living with a single parent were the risk factors significantly associated with ADHD, family, twin, and adoption studies have suggested that ADHD is as highly heritable as schizophrenia and bipolar disorders (72). The role of genes has also been reported in molecular genetic studies (72). There is a neurocognitive difference in neurophysiological profile and neuropsychological testing with each subtype of ADHD (72).

Other evidence suggests that ADHD is the result of brain differences (abnormalities in size, maturation, and activities in the region of the brain involved in executive function and self-regulation). Less electrical activity in ADHD-affected regions of the brain, decreased blood flow, lower metabolism of glucose in the frontal regions of the brain, and decreased activity levels in the frontal and basal ganglia of the brain have been reported in children with ADHD compared with non-ADHD children (72, 79, 80). This implies that environmental factors for genetic exposure for ADHD might be preventable and need to be audited.

## Limitation of the study

The high heterogeneity of the included studies is the limitation of this study. This may affect its direct use for decision-making. Furthermore, because this study only looked at cross-sectional papers, it was impossible to track the participants and measure their symptoms over time. Another limitation of this study is that most of the included studies were from one region (Oromia); the current estimates of the pooled prevalence of ADHD may be underestimated or overestimated in Ethiopia. The use of varied data collection tools in the included research may have an impact on generalizing the findings. In addition, the study’s lack of PROSPERO registration may result in double publications in databases.

## Conclusion

In conclusion, the prevalence of ADHD is within the range of its global estimates. The result showed that one out of twelve children in Ethiopia had ADHD. Being 6–12 years old, being male, and living with a single parent were significant predictors of ADHD.

## Recommendation

As most studies with large sample sizes reported similar prevalences of ADHD, a nationwide study with a large sample size might be important. The results of this investigation corroborate previous research and highlight the importance of planning and policymaking in screening and controlling the day-to-day and long-term consequences of ADHD in children and adolescents in Ethiopia. Most studies conducted with ICD-10 criteria reported a relatively small prevalence of ADHD. There have been no reported Ethiopian studies that have applied ICD-10 diagnostic criteria. Thus, a comparison study using the two criteria (DSM-V and ICD-10) is advisable. Furthermore, future research should look into the prevalence of ADHD in different age groups and how it affects their personal and social lives. Finally, ADHD prevalence is frequently associated with genetics, diet, and environment. As most of the included studies were from the Oromia region, a nationwide study with an adequate sample size is important.

## Data Availability

The original contributions presented in the study are included in the article/Supplementary Material, further inquiries can be directed to the corresponding author, contact address: molla2ab@gmail.com.
