# Supplementary material for: The prevalence of attention-deficit hyperactivity disorder and its associated factors among children in Ethiopia, 2024: a systematic review and meta-analysis
Source: Front Child Adolesc Psychiatry. 2024 Aug 23;3:1425841. doi: 10.3389/frcha.2024.1425841 (PMC11751470; doi:10.3389/frcha.2024.1425841)
Supplement: Supplementary file 1 [file Table1.docx]

**Quality assessment**

**JBI’s critical appraisal Checklist for cross-sectional studies**

| Primary studies | JBI’s critical appraisal questions | | | | | | | | Quality/8 | Included |
| --- | --- | --- | --- | --- | --- | --- | --- | --- | --- | --- |
|  | Q1 | Q2 | Q3 | Q4 | Q5 | Q6 | Q7 | Q8 |  |  |
| Ashenafi, Y., et al (2001) | Y | Y | Y | Y | Y | Y | Y | Y | 8 | √ |
| Mulu, G. B., et al(2022) | Y | Y | Y | Y | Y | N | N | Y | 6 | √ |
| Lola, H. M., et al (2019) | Y | Y | Y | Y | Y | Y | Y | Y | 8 | √ |
| Benti, M., et al (2021) | Y | Y | Y | Y | Y | N | Y | Y | 7 | √ |
| Tiruneh, F. et al (2015) | N | Y | Y | Y | Y | N | Y | Y | 6 | √ |
| Aliye, K., et al (2023) | Y | Y | Y | Y | Y | N | Y | Y | 7 | √ |

| Q1. Were the criteria for inclusion in the sample clearly defined? |  |  |  |  |
| --- | --- | --- | --- | --- |
| Q2. Were the study subjects and the setting described in detail? |  |  |  |  |
| Q3. Was the exposure measured in a valid and reliable way? |  |  |  |  |
| Q4. Were objective, standard criteria used for measurement of the condition? |  |  |  |  |
| Q5. Were confounding factors identified? |  |  |  |  |
| Q6. Were strategies to deal with confounding factors stated? |  |  |  |  |
| Q7. Were the outcomes measured in a valid and reliable way? |  |  |  |  |
| Q8. Was appropriate statistical analysis used? |  |  |  |  |

**NB**. Y: Yes, N: No, U: Unclear, Q: Question. The overall score is calculated by counting the number of Y’s in each row.
